# A Tuba/Cdc42/Par6A complex is required to ensure singularity in apical domain formation during enterocyte polarization

**DOI:** 10.1371/journal.pone.0207159

**Published:** 2018-11-08

**Authors:** Lucas J. M. Bruurs, Mirjam C. van der Net, Susan Zwakenberg, Fried J. T. Zwartkruis, Johannes L. Bos

**Affiliations:** Oncode Institute, Center for Molecular Medicine, University Medical Center Utrecht, Utrecht University, Utrecht, The Netherlands; NCMLS, Radboud University Nijmegen Medical Center, NETHERLANDS

## Abstract

Apico-basal polarity establishment is a seminal process in tissue morphogenesis. To function properly it is often imperative that epithelial cells limit apical membrane formation to a single domain. We previously demonstrated that signaling by the small GTPase Cdc42, together with its guanine nucleotide exchange factor (GEF) Tuba, is required to prevent the formation of multiple apical domains in polarized Ls174T:W4 cells, a single cell model for enterocyte polarization. To further chart the molecular signaling mechanisms that safeguard singularity during enterocyte polarization we generated knockout cells for the Cdc42 effector protein Par6A. Par6A loss results in the formation of multiple apical domains, similar to loss of Cdc42. In Par6A knockout cells, we find that active Cdc42 is more mobile at the apical membrane compared to control cells and that wild type Cdc42 is more diffusely localized throughout the cell, indicating that Par6A is required to restrict Cdc42 signaling. Par6A, Cdc42 and its GEF Tuba bind in a co-immunoprecipitation experiment and they partially colocalize at the apical membrane in polarized Ls174T:W4 cells, suggesting the formation of a trimeric complex. Indeed, in a rescue experiment using Par6A mutants, we show that the ability to establish this trimeric complex correlates with the ability to restore singularity in Par6A knockout cells. Together, these experiments therefore indicate that a Tuba/Cdc42/Par6A complex is required to ensure the formation of a single apical domain during enterocyte polarization.

## Introduction

The establishment of functionally distinct apical and basolateral domains in epithelial cells is a seminal step in the organization of epithelial tissues. An important feature of most polarized cells is that polarization is restricted to the formation of a single apical domain. The small GTPase Cdc42 has an evolutionary conserved pioneering function in the establishment of an apical membrane and its signaling has been implicated in safeguarding singularity in yeast cell polarization [1].

In the context of an intact epithelial monolayer, cell-cell junctions separate the apical and basolateral domain and thereby ensure the formation of a single apical domain. Nevertheless, epithelial cell lines that polarize in the absence of cell-cell junctions rarely form more than one apical domain [2, 3], indicating that singularity in apical membrane formation is ensured in a junction-independent manner. Using Ls174T:W4 cells, a single cell model for enterocyte polarization in which polarity is induced by forced activation of LKB1 [2], we previously demonstrated that Cdc42 signaling is required to ensure the formation of a single apical domain [4]. For this, Cdc42 activity and mobility at the nascent apical plasma membrane is strictly regulated by the Cdc42-specific GEF Tuba [5]. However, it remains unclear how the GEF Tuba can control Cdc42 mobility and which effector(s) Cdc42 engages to ensure the formation of a single apical domain.

Here, we show that the Cdc42 effector Par6A is required for singularity in enterocyte polarization. We show that Par6A limits the mobility of active Cdc42 at the nascent apical membrane and that Par6A can form a complex with Cdc42 and Tuba. Therefore, this work reveals that a Tuba/Cdc42/Par6A complex regulates the formation of a single apical domain during cell polarization.

## Materials & methods

### Cell culture and plasmids

Ls174T:W4 cells [2] were cultured in RPMI1640 (Sigma) supplemented with 10% FBS (Sigma) and antibiotics. For the induction of polarization, cells were trypsinized and transferred to medium containing 1 μg/ml doxycycline (Sigma) for at least 16h. For transient expression of DNA constructs, cells were transfected using XtremeGene9 (Roche) according to the manufacturer’s guidelines.

pK-myc-Par6c (Addgene plasmid # 15474) was a provided by Ian Macara and served as a template for to generate pDEST-Par6A using In-Fusion cloning (Clontech). To generate Par6A(ΔCRIB), in which amino acids 134 to 151 were deleted, two Par6A PCR fragments were generated (one upstream of the CRIB domain and one downstream) containing a compatible overhang and assembled using In-Fusion. The I133A, S134A mutation was introduced using a similar strategy. For the PB1 deletion mutant, the N-terminal 95 amino acids were deleted. N-terminal fusion proteins of Par6A and these mutants were generated using Gateway cloning (Invitrogen). N-terminal fusions of Cdc42, Tuba and EBP50 were generated using Gateway cloning.

### Antibodies

The following antibodies were used for Western blotting: rabbit anti-Par6A (Sigma Prestige, 1:1000), anti-HA (12CA5, Roche, 1:10000), mouse anti-GFP (clones 7.1 and 13.1, Roche, 1:5000), mouse anti-V5 (Invitrogen, 1:5000), mouse anti-GAPDH (6C5, Millipore, 1:5000) and mouse anti-β-Catenin (BD biosciences, 1:5000).

### Generation of Par6A knockout Ls174T:W4 cells

Par6A knockout Ls174T:W4 cells were generated using CRISPR/Cas9-mediated gene disruption as previously reported [4]. Briefly, Ls174T:W4 cells were transfected with pSpCas9(BB)-2A-GFP (PX458), encoding an sgRNA (5’- GCGGGCGGTGCACCAGATCC-3’) targeting the exon encoding the PB1 domain of Par6A. GFP-positive cells were sorted and clonally expanded. Genomic DNA of candidate clones was isolated and sequenced using the following primers: FW_5’- CGAGGTGAAGAGCAAAGTAAG-3’ and RV_5’-CAGAGAGTTGGAGGCAAAAG-3’. Absence of Par6A protein was confirmed by Western blotting.

### Live cell imaging and determining apical membrane mobility of Dendra-Cdc42(G12V)

At least two days after transfection, cells were trypsinized and cells were seeded on glass bottom dishes (WillCo Wells) in doxycycline-containing medium. Cells were imaged in Leibovitz’s L-15 medium (Invitrogen) at 37°C using an Axioskop2 LSM510 or LSM880 scanning confocal microscope (Zeiss) with a 63x magnification oil objective (PLAN Apochromat, NA 1.4) using Zen image acquisition software. Apical enrichment of YFP-Cdc42 was quantified by making a line scan through the apical and basal membrane using ImageJ. From this the ratio between average apical and basal membrane pixel intensities was determined. Average enrichment ratios were compared using independent samples t-test with a p-value <0.05 as a cutoff for significance. Average line plot was generated by determining the average normalized signal intensities over a 14 μm section where the brush border was positioned in the center.

Apical mobility of Dendra-Cdc42(G12V) was determined as previously described [5]. In short, transfected cells were polarized and imaged with a Leica SP8x microscope equipped with a temperature- and CO_2_-controlled chamber using a 63x oil objective (HC PL APO 63x/1.40) with Leica LAS AF image acquisition software. After a local 405nm laser pulse, cells were imaged at 1.5 sec/frame rate. The ratio of the average red signal intensity in the brush border and the whole cell was determined for every time point and fitted using Matlab with the general formula: f(x) = a * e^(-b*x) + c. An average half-life was determined from the fitted curve and expressed with the 95% CI of the fit. Immobile fractions were determined from the plateau value of the fit (i.e. “c”) and expressed with the 95% CI of the fit.

### Co-immunoprecipitation

Transfected HEK293T cells were scraped in cold lysis buffer (1% Nonidet P-40 substitute; 10% glycerol; 50 mM Tris-HCl pH 7.4; 2.0 mM MgCl_2_; 200 mM NaCl; protease and phosphatase inhibitors) and cleared by centrifugation. Cleared lysates incubated with agarose beads coupled to GFP-binding protein (GBP) for 1h at 4°C while rotating. Beads were washed three times with lysis buffer and bound proteins were eluted in sample buffer. 5% Of the cleared lysate was loaded for the total lysate.

For the quantification of relative binding, the background-corrected band intensities of V5-Par6 signal was divided by the YFP-Tuba signal and normalized to the condition in which most binding observed. Average relative bindings were compared using paired samples t-test with a p-value <0,05 as a cutoff for significance.

## Results

**Fig 1:**
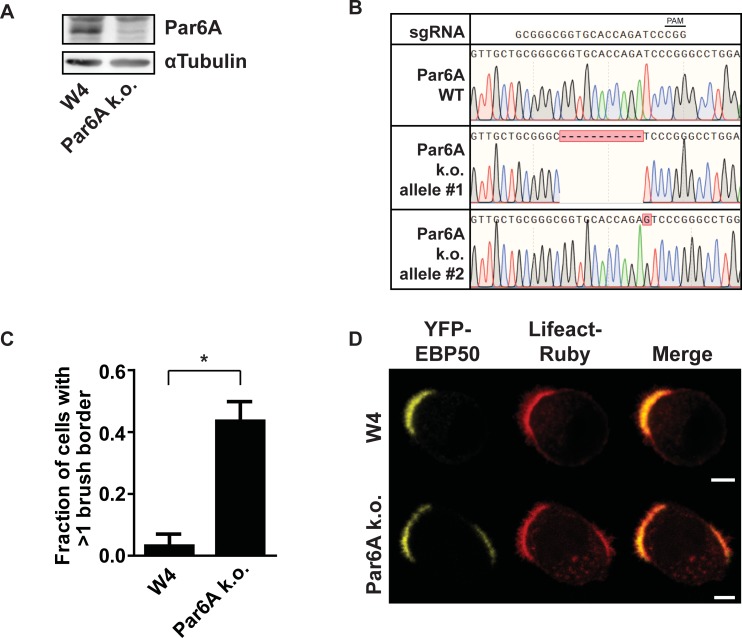
Par6A k.o. Ls174T:W4 cells form multiple brush borders upon polarization. A) Western blot of Ls174T:W4 and Par6A k.o. cell lysate probed with Par6A and αTubulin antibody. B) Alignment of sequence traces of wild type Par6A and the two edited alleles of Par6A k.o. cells. The top row shows the short guide RNA (sgRNA) used for Cas9 mediated gene disruption and indicated in that sequence is the protospacer adjacent motif (PAM) sequence. C) Quantification of the fraction of cells that form more than one brush border as determined by YFP-EBP50 and lifeact-ruby distribution in polarized Ls174T:W4 or Par6A k.o. cells. Error bars indicate sd (n = 3, >75 per experiment) * *p* < 0.05 using independent samples t-test. D) Representative images of polarized Ls174T:W4 and Par6A k.o. cells expressing YFP-EBP50 and lifeact-ruby. Scale bars indicate 5μm.

In search of a Cdc42 effector protein that ensures singularity in apical membrane specification we focused on the scaffold protein Par6, which contains a CRIB-PDZ module via which it can specifically interact with GTP-loaded Cdc42 and which has a well-documented role in polarity establishment [6–8]. Since Par6A is expressed in intestinal epithelial cells and is predominantly located at the cell cortex where active Cdc42 is located, we generated Ls174T:W4 cells in which the *PARD6A* gene was destroyed using CRISPR-Cas9 [7, 9]. We produced a cell line in which Par6A expression was lost (further referred to as Par6A k.o.), as detected by Western blotting using a Par6A specific antibody (Fig 1A). Genotyping confirmed that both alleles of the *PARD6A* gene were edited, containing an 11bp deletion and 1bp insertion, which both resulted in the introduction of a premature stop codon (Fig 1B).

When polarization was induced in Par6A k.o. cells, a large fraction of cells formed multiple patches of actin-rich microvilli as judged by distribution of the actin marker Lifeact-Ruby (Fig 1C). To demonstrate that these actin-rich patches indeed represent an apical brush border, we expressed the brush border marker YFP-EBP50 [10]. Upon polarization of control Ls174T:W4 cells YFP-EBP50 is restricted to the actin cap that constitutes the apical brush border (Fig 1D). In Par6A k.o. cells, the actin-rich caps are positive for YFP-EBP50 indicating that these patches are genuine apical brush borders (Fig 1D). Therefore, Par6A loss phenocopies Cdc42 loss in Ls174T:W4 cells and results in the formation of multiple apical domains.

**Fig 2:**
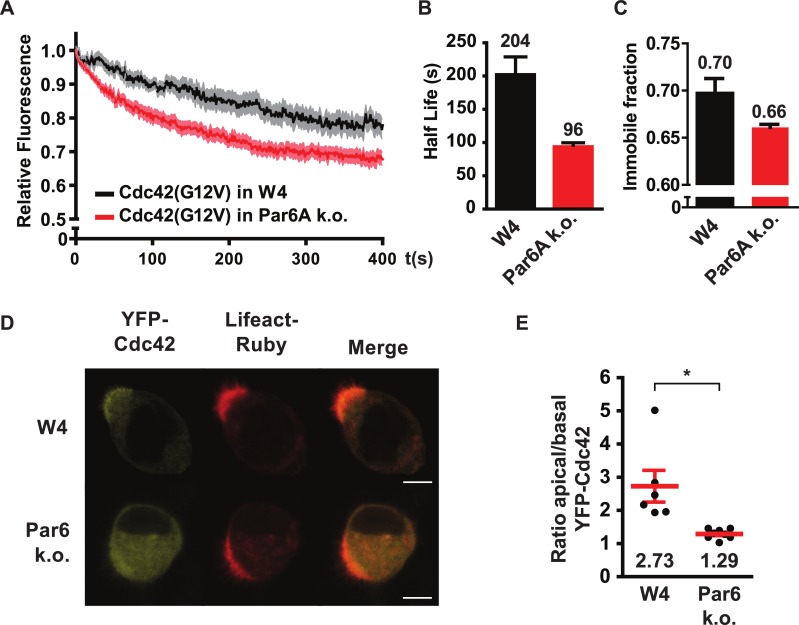
Apical mobility of Cdc42(G12V) is increased in Par6A k.o. cells. A) Average normalized fluorescence decay traces of Dendra-Cdc42(G12V) in polarized Ls174T:W4 cells (black line, n = 12) and Par6A k.o. cells (red line, n = 17). Light area indicates sem. B) Eviction half-lifes for Dendra-Cdc42(G12V) in polarized Ls174T:W4 cells and Par6A k.o. cells determined from average decay traces in A using curve fitting. Error bars indicate 95% confidence interval of the fit. C) Immobile fractions for Dendra-Cdc42(G12V) in polarized Ls174T:W4 cells and Par6A k.o. cells determined from average decay traces in A using curve fitting. Error bars indicate 95% confidence interval of the fit. D) Representative images of polarized Ls174T:W4 and Par6A k.o. cells expressing YFP-Cdc42 and lifeact-ruby. Scale bars indicate 5μm. E) Quantification of apical enrichment of YFP-Cdc42 signal intensity in polarized Ls174T:W4 and Par6A k.o. cells. Red lines indicate the average and s.e.m. (W4: n = 6; Tuba k.o. n = 8). * *p* < 0.05 using independent samples t-test.

**Fig 3:**
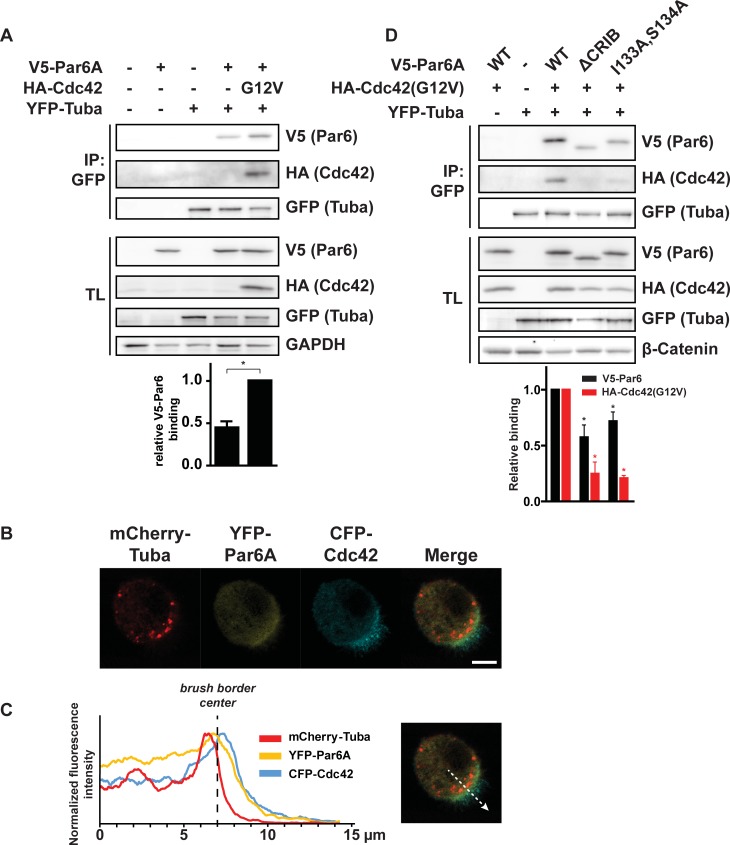
Par6A, Cdc42 and Tuba co-immunoprecipitate and colocalize at the apical domain in polarized Ls174T:W4 cells. A) Co-immunoprecipitation of V5-Par6A, HA-Cdc42(G12V) and YFP-Tuba in HEK293T cells. Bottom: quantification of relative V5-Par6 binding to YFP-Tuba in the presence and absence of HA-Cdc42(G12V). Error bars indicate s.e.m. (n = 3) * *p* < 0,05 using paired samples t-test. B) Localization of mCherry-Tuba, YFP-Par6A and CFP-Cdc42 in polarized Ls174T:W4 cells. Scale bar indicates 5μm. C) Left: Plot of average normalized fluorescence intensities of mCherry-Tuba, YFP-Par6A and CFP-Cdc42 in an apical section of 6 cells. Right: example of apical section used to generate average line scan. D) Co-immunoprecipitation of V5-Par6A, either WT, ΔCRIB or I133A,S134A with HA-Cdc42(G12V) and YFP-Tuba in HEK293T cells. IP = immunoprecipitation, TL = total lysate, WT = wild type. Bottom: quantification of relative V5-Par6 and HA-Cdc42(G12V) binding to YFP-Tuba. Error bars indicate s.e.m. (n = 4) * *p* < 0,05 using paired samples t-test.

We previously showed that Cdc42 mobility at the apical membrane is highly regulated via a RhoGDI-dependent and Tuba-dependent mechanism and that this regulation is critical for Cdc42 function [5]. To test whether Par6A contributes to the regulation of Cdc42 mobility we determined the mobility of constitutively active Cdc42(G12V) at the apical membrane of polarized Ls174T:W4 cells and Par6A k.o. cells. For this we locally photoconverted Dendra-fused Cdc42(G12V) in the brush border and followed its subsequent loss from the converted region as a consequence of lateral diffusion [5]. We find that the apical mobility of Cdc42(G12V) is higher in Par6A k.o. cells compared to control cells (Fig 2A & 2B). In addition, the immobile fraction of Dendra-Cdc42(G12V) molecules was lower in Par6A k.o. cells (Fig 3C). These findings therefore indicate that Par6A contributes to the regulation of Cdc42 mobility by immobilizing active Cdc42 at the apical membrane. To determine the consequence of Par6A loss on Cdc42 localization we determined the apical enrichment of YFP-Cdc42 in polarized Ls174T:W4 cells and Par6A k.o. cells. We find that in Par6A k.o. cells YFP-Cdc42 is more diffusely localized and is less concentrated at the apical membrane (Fig 2D & 2E). Together, these findings demonstrate that loss of Par6A-dependent immobilization of active Cdc42 is required to enrich Cdc42 at the apical membrane.

How can Par6A induce the immobilization of active Cdc42? Since Par6A functions as a scaffold protein, we hypothesized that Par6A may immobilize active Cdc42 by making it part of a multiprotein complex. Because we previously demonstrated that, similar to Par6A, the Cdc42GEF Tuba is also required for the immobilization of constitutively active Cdc42(G12V) [5], we tested whether Tuba, Par6 and Cdc42 could form a complex. Indeed, we find that V5-Par6A binds YFP-Tuba and that this binding is increased in the presence of active HA-Cdc42(G12V) (Fig 3A). Cdc42 itself also co-immunoprecipitates with YFP-Tuba and V5-Par6A, indicating that it stably associates with this GEF/effector complex.

We next assessed the localization of these proteins in polarized Ls174T:W4 by life cell imaging. Although the distribution is not identical, all three proteins have an overlapping localization at the apical domain in polarized Ls174T:W4 cells, compatible with the notion that they form a trimeric complex (Fig 3B & 3C).

To further test how active Cdc42 can promote the formation of a Tuba/Par6 complex we tested the ability of Par6A mutants that are unable to bind active Cdc42 to form a trimeric complex. We find that deletion of the whole CRIB domain and mutation of two residues required for Cdc42-GTP binding largely abrogates HA-Cdc42 binding (Fig 3D) [7]. In addition, the binding of these Par6A mutants to YFP-Tuba is reduced, compatible with the notion that Cdc42 is stabilizing the complex. This residual binding may indicate that Par6A directly binds YFP-Tuba, but, alternatively, endogenous Cdc42 may be responsible for this binding.

**Fig 4:**
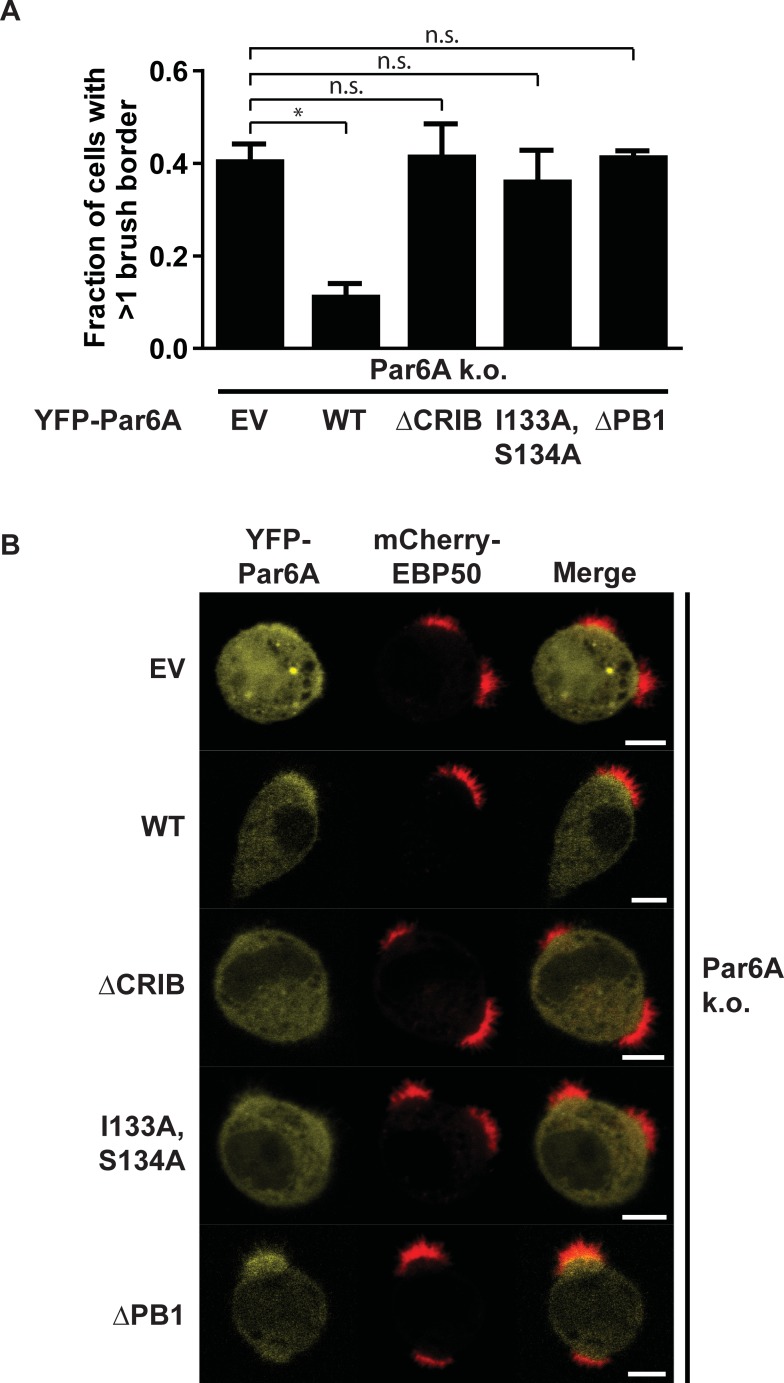
Cdc42-binding defective and PB1 deletion mutants of Par6A can not rescue singularity in apical membrane in Par6A k.o. cells. A) Quantification of the fraction of cells that form more than one brush border as determined by mCherry-EBP50 distribution in Par6A k.o. cells expressing YFP empty vector (EV) or YFP-Par6A(WT), YFP-Par6A(ΔCRIB), YFP-Par6A(I133A,S134A) or YFP-Par6A(ΔPB1). Error bars indicate sd (n ≥ 3, >85 cells in total per condition) * *p* < 0.05 using independent samples t-test. n.s. *p* > 0.05 using independent samples t-test. B) Representative images of polarized Par6A k.o. cells expressing YFP empty vector (EV), YFP-Par6A(WT), YFP-Par6A(ΔCRIB), YFP-Par6A(I133A,S134A) or YFP-Par6A(ΔPB1) in combination with mCherry-EBP50. Scale bars indicate 5μm.

To assess whether the ability to bind active Cdc42 affects Par6A localization and function we re-expressed wild type and the Cdc42-binding mutants of Par6A in Par6A k.o. cells and assessed their ability to restore singularity in apical domain formation. We find that wild type Par6A can largely rescue the singularity defect, indicating that this phenotype is not the result of an off-target effect of the CRISPR procedure (Fig 4A & 4B). In contrast, expression of the Cdc42-binding mutants YFP-Par6A(ΔCRIB) and YFP-Par6A(S133A,I134A) did not restore singularity in apical membrane formation (Fig 4A & 4B). In addition, whereas wild type Par6A is enriched at the apical domain, such apical accumulation is not observed for either mutant (Fig 4B). This therefore demonstrates that Par6A requires Cdc42 to become enriched at the apical membrane and that the interaction with Cdc42 is necessary to ensure singularity in apical membrane specification.

The further signaling pathway engaged by Par6A to regulate singularity is still unclear. Atypical PKCs (ι/λ and ζ) interact with Par6 and in part mediate the effects of Par6 during polarity establishment [7]. To get an indication whether aPKCs may contribute to the formation of a single apical domain, we expressed in Par6A k.o. cells a Par6A mutant which is unable to bind aPKCs (YFP-Par6A(ΔPB1)) [11]. We find that this mutant can not rescue the Par6A k.o. phenotype suggesting that aPKCs are involved in the formation of a single apical domain (Fig 4A & 4B). However, to elucidate this process in detail requires further investigation.

## Discussion

Here we demonstrate that loss of the Cdc42 effector protein Par6A results in the formation of multiple apical domains in polarized Ls174T:W4 cells, similar to loss of Cdc42 [4]. This indicates that Par6A is the effector of Cdc42 to ensure singularity in apical membrane formation. Additionally, we demonstrate that Tuba, Cdc42 and Par6A can form a trimeric complex under overexpression conditions and that they have an overlapping localization at the apical domain. We find a correlation between the ability of Par6A to support the formation of this trimeric complex and the ability to restore singularity in Par6A k.o. cells.

The notion that a Tuba/Cdc42/Par6A complex regulates singularity is based on three observations. Firstly, we find that Par6A and the Cdc42-specific GEF Tuba interact and that this interaction depends in part on the ability of Par6A to bind active Cdc42. Since GEFs and effectors bind to the same region of a small GTPase, the binding of Tuba, via its catalytic DH domain, and the binding Par6A, via its CRIB domain, to Cdc42 is mutually exclusive. The finding that Tuba, Cdc42 and Par6A co-immunoprecipate is therefore most likely explained by an intrinsic affinity of Par6A towards Tuba which is increased by Cdc42 binding. In support of this, we find that Par6A binds weakly to Tuba in the absence of (exogenous) Cdc42 (Fig 3A) and that Cdc42-binding defective Par6A mutants do not completely lose the ability to bind Tuba (Fig 3D). Secondly, both Tuba [5] and Par6A (Fig 2) are required to immobilize Cdc42 at the plasma membrane. We demonstrated previously that immobilization of Cdc42 at the apical plasma membrane is required to safeguard singularity in apical membrane formation [4]. Thirdly, Par6A mutants that are unable to interact with Cdc42 and as a consequence do not support the formation of a Cdc42/Tuba/Par6A complex are unable to restore singularity in apical membrane formation when introduced in Par6A k.o. cells. Therefore these experiments identify Par6A as a Cdc42 effector that ensures singularity in polarization and provide evidence that the formation of a Cdc42/Tuba/Par6A complex is important in this process.

The existence of a GEF/effector complex regulating Cdc42 signaling is not without precedence and bears striking resemblance to the Cdc24/Bem1 complex that regulates Cdc42 during yeast cell polarization [12, 13]. During yeast cell polarization, this complex establishes a positive feedback loop for the production of active Cdc42 at the incipient bud site [13]. This positive feedback loop is critical to allow symmetry breaking and for the establishment of a single cluster of active Cdc42 [14–16]. Therefore, it is tempting to speculate that a Tuba/Cdc42/Par6A complex may perform a similar function during epithelial cell polarization.

Par6A is a scaffold protein that engages in multiple interactions with important regulators of cell polarity, including Par3 [8], aPKC [11, 17], Pals1 [18] and Lgl [19]. We now show that the Cdc42-specific GEF Tuba is able to interact with Par6A in manner that is largely dependent on active Cdc42. Since Tuba is implicated in a variety of polarity processes that depend on Cdc42 and Par6, this interaction may not only be of relevance for singularity enforcement but also for junction formation/stability [20, 21] and spindle orientation [22, 23]. What other proteins are part of the Tuba/Cdc42/Par6 complex and how the composition of this complex is regulated requires further investigation. The finding that Par6A requires its N-terminal PB1 domain to enforce singularity indicates a role for aPKCs in this process, but additional Par6 binding partners may contribute to safeguard singularity in polarization as well.

In summary, we reveal that Par6A is a Cdc42 effector that is required to ensure the formation of a single apical domain during enterocyte polarization. We show that Par6A can form a complex with Cdc42 and its GEF Tuba and that the formation of this complex contributes to the organization of Cdc42 signaling to regulate singularity. This work thus provides new insights in the molecular mechanisms of Cdc42 signaling during cell polarization.
